# Deep Learning Methods in Predicting Gene Expression Levels for the Malaria Parasite

**DOI:** 10.3389/fgene.2021.721068

**Published:** 2021-09-22

**Authors:** Tuan Tran, Banafsheh Rekabdar, Chinwe Ekenna

**Affiliations:** ^1^Department of Computer Science, University at Albany, Albany, NY, United States; ^2^Department of Computer Science, Southern Illinois University, Carbondale, IL, United States

**Keywords:** genes expression, genes profile, recurrent neural network, *Plasmodium falciparum*, machine learning, malaria, spiking neural network

## Abstract

Malaria is a mosquito-borne disease caused by single-celled blood parasites of the genus *Plasmodium*. The most severe cases of this disease are caused by the *Plasmodium* species, *Falciparum*. Once infected, a human host experiences symptoms of recurrent and intermittent fevers occurring over a time-frame of 48 hours, attributed to the synchronized developmental cycle of the parasite during the blood stage. To understand the regulated periodicity of *Plasmodium falciparum* transcription, this paper forecast and predict the *P. falciparum* gene transcription during its blood stage life cycle implementing a well-tuned recurrent neural network with gated recurrent units. Additionally, we also employ a spiking neural network to predict the expression levels of the *P. falciparum* gene. We provide results of this prediction on multiple genes including potential genes that express possible drug target enzymes. Our results show a high level of accuracy in being able to predict and forecast the expression levels of the different genes.

## 1. Introduction

According to the World Health Organization, malaria has established itself as one of the leading causes of death in developing countries concentrated within the tropics and subtropics. In 2017, there were an estimated 219 global million malaria cases, where the majority of these cases were found in Sub-Saharan Africa and Southeast Asia. While there is a projected downward trend in expected malaria cases, the high mutational capacity of the *Plasmodium* parasite coupled with its changing metabolism makes the development of new effective drug treatments a continuous problem.

As an apicomplexan, *Plasmodium falciparum* do not have a single stable life stage. Instead, they periodically transition between several intermediate stages within a complete life cycle. Beginning with the injection of infectious sporozoites from the mosquito gut to the human circulation, the parasite migrates to hepatocytes where they consume intracellular content and rapidly proliferate, preparing themselves for erythrocytic invasion post cell lysis. Once within the erythrocytes, the parasite adjusts itself to its immediate environment by transitioning between the three distinct stages: trophozoite, merozoite, schizont. Over a general time frame of 48 h, it cycles through these stages causing a series of synchronized mass erythrocyte destruction and invasion, giving rise to the clinical symptoms of intermittent fever associated with malaria [Centers for Disease Control and Prevention (CDC), 2017]. Corresponding to the requirements of rapid cloning during this stage, separate studies have measured a significant increase in glucose uptake, parasitic growth rate, hemoglobin degradation, and transcriptional activity (Read et al., 2019). For these reasons, the blood stage of *P. falciparum* remains an area of high interest for antimalarial development.

Currently, one of the primary open questions in *Plasmodium* biology is how the parasite maintains precise control of gene expression during these stages (Read et al., 2019). It is well-established that gene expression is a highly regulated process, allowing the parasite to perform the necessary biological functions at a given developmental stage. Considering the role of mRNA as an intermediate product between transcribed DNA and the translated protein, precise time-dependent gene expression profiles can be used to identify stage-specific metabolic genes as a means to better understand how the Plasmodium metabolism changes relative to time. From the central dogma, it can be understood that transcribed DNA precedes the translation of a protein. With high concentrations of distinct mRNA stands within a cell, high concentrations of the corresponding protein will be expected. Despite conflicting ideas about the transcriptome-proteome interaction, it has been shown that there is still a positive association between mRNA and protein levels (Bozdech et al., 2003). Overall, there is an undeniable relationship between mRNA and protein abundances which lends itself toward understanding the relative availability of certain enzymes at a given point in development based on gene expression data.

**Figure 1:**
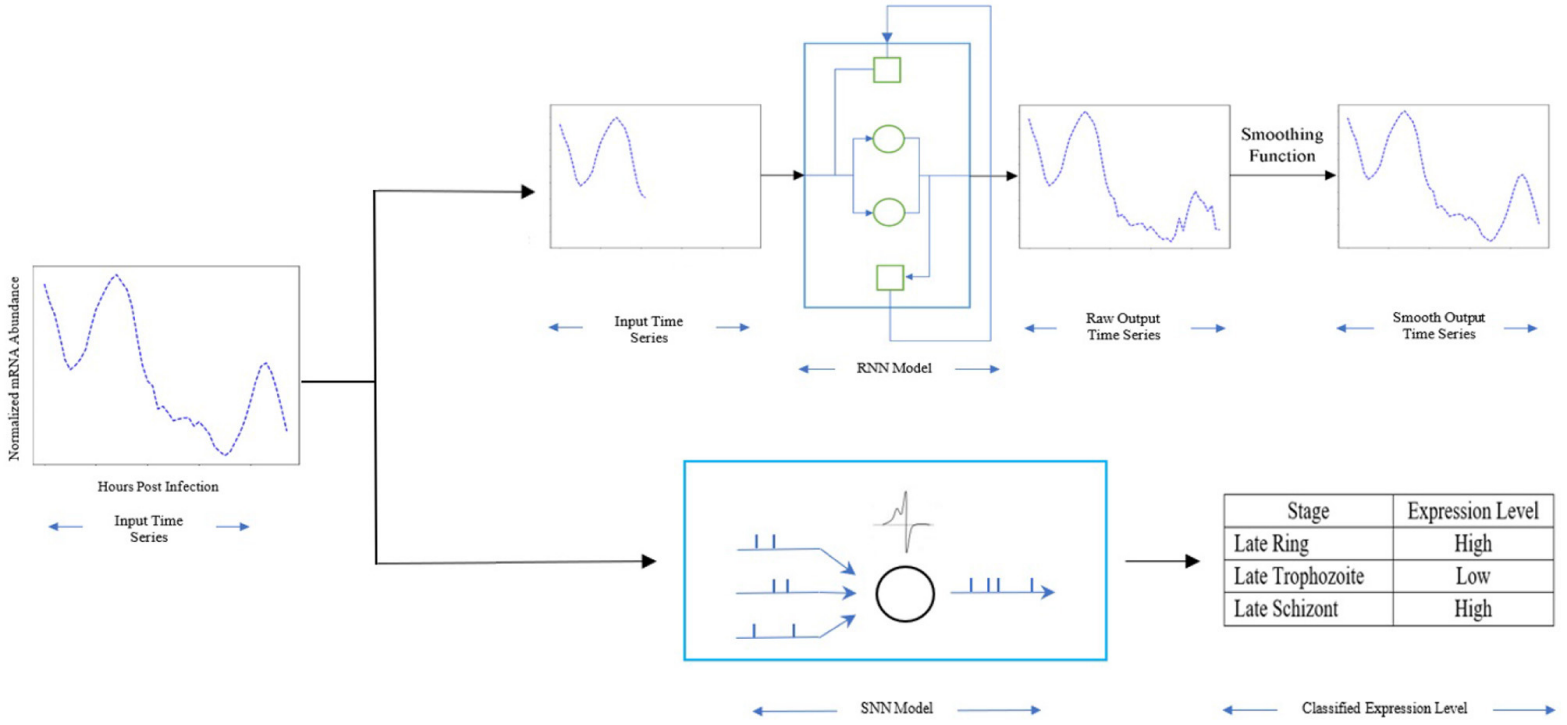
The method overview: Light blue rectangle represents our RNN model with the gated units as cycles, the hidden units as squares, the dark blue rectangle represents our SNN model with the spiking neuron as cycles. The input is the mRNA abundance time series of PF3D7_1337500. The early stage time series is used for RNN, the whole time series is used for SNN. The output of RNN is the mRNA abundance time series during its life cycle in blood stage, the output of SNN is the expression level for multiple stages.

In this work, we estimate the expression profile of genes associated with the essential enzymes thus providing a tool to help reduce the timeline needed to create these profiles. To achieve that, we proposed a recurrent neural network model to forecast the mRNA abundance throughout the 48 h post-infection (hpi) in the intraerythrocytic developmental cycle (IDC) for the malaria parasite *P. falciparum*. Figure 1 shows an overview of our approach. First, a Recurrent Neural Network (RNN) is implemented, trained, and validated using 80% of our dataset. Then the RNN is used to forecast a time series of gene expression for prior gene expression information. Finally, a function is applied to smooth the forecast time series. Additionally, with the mRNA abundance throughout the 48 h post-infection, we employed a spiking neural network (SNN) to profile the gene expression level (low or high) for multiple stages in the intraerythrocytic developmental cycle. The SNN is trained and validated using part of the dataset and then is used to predict the genes expression level based on the mRNA abundance throughout the 48 h post-infection. By using a quite extensive dataset of gene expression for *P. falciparum* from current literature with multiple potential drug targets, the results in this paper show an accurate prediction and forecast of the mRNA abundance for the next stage of the parasite, and the genes expression level during the 48 h IDC. Overall, our approach provides precise estimations, was able to accurately provide the trend and expression level in the gene profiles when compared with experimental data.

## 2. Related Work

Amongst the multitude of biological processes occurring within a cell, metabolism is considered to be among the most researched. Specifically, antimalarial development focuses on identifying essential metabolic components that can serve as drug targets (Carey et al., 2017). Multiple studies approach this idea by constructing a network model of the parasitic metabolism, relevant to the specific stages (Dholakia et al., 2015; Phaiphinit et al., 2016). Knowing that the parasite employs different metabolic components for each stage, it is possible to determine effective drug targets based on which set of enzymes are most significant at a given developmental stage.

Focusing on the relationship between the developmental stage and transcriptional activity, several studies have observed the variation in mRNA abundance through microarray measurements at sustained time intervals. For instance, Painter et al. (2018) were able to produce a genome-wide mRNA abundance profile at a 1-h resolution for the complete 48 h of the intraerythrocytic stage. This representation shows the cascading nature of *P. falciparum* gene expression, where a single gene is highly expressed for a discrete segment of time and is followed by a succession of genes each with their segment of expression. Moreover, Mamoun et al. (2001) showed that histone methylation is more heavily related to the schizont stage which may correspond to reduced gene expression profiles during this stage. Ultimately, their work points toward an inherent pattern in gene expression data which we capture in this paper through machine learning techniques used to identify key enzymes and peak gene expression at a given stage.

Recently, deep learning models, such as Convolutional Neural Network (CNN) and Recurrent Neural Network (RNN), have seen increased usage in biology and medical applications. Tavanaei et al. (2017) proposed CNN for predicting tumor suppression genes and proto-oncogenes. Additionally, Kelley et al. (2016) learned the functional activity of DNA sequences from genomics data by applying CNNs. Multiple machine learning models were applied to discriminate between high-expression and low-expression genes, focusing on three distinct stages of the red blood cell phase of the Plasmodium life cycle (Read et al., 2019). Moreover, Singh et al. (2016) developed a unified discriminate framework using a deep CNN to classify gene expression using histone modification data.

Various time series methods such as Auto-regressive Moving Average (Buendia and Solano, 2015; Ebrahimi et al., 2017); Simple Exponential Smoothing (Luo et al., 2017); Holt-Winters Exponential Smoothing (Ghaffari et al., 2018) have been widely applied to electronic health records. Recently, a Gated Recurrent Unit (GRU) was introduced to handle missing values in multivariate time series data (Che et al., 2018). A Time-Aware Long Short term memory (LSTM) model was proposed to handle irregular time intervals in longitudinal patient records to capture the progression patterns for Parkinson's disease (Baytas et al., 2017). In this work, we developed an RNN using GRU to forecast the gene expression profile for three different stages of the *P. falciparum* parasite during the blood stage life cycle.

Spiking Neural Networks (SNN) are also being used in various applications. Specifically, Kasabov (2014) demonstrated that SNNs are suitable for the creation of a unifying computational framework for learning and understanding of various spatio-and spectro-temporal brain data. Moreover, Antelis et al. (2020) showed the capabilities of SNN in the recognition of motor imagery tasks from electroencephalography signals. In Morro et al. (2017), they presented a smart stochastic spiking neural network architecture to efficiently enhance the time-consuming process for virtual screening. Finally, Vellappally et al. (2018) applied the xeno-genetic spiking neural network for examining dental problems and facilitating oral health maintenance. In this work, we developed an SNN to classify the gene expression levels for different stages of the *P. falciparum* parasite during the blood stage life cycle.

## 3. Methodology

### 3.1. RNN Architecture

RNNs, are fully connected neural networks with recurrent connections in their hidden layers, specialize in processing a sequence of values (Goodfellow et al., 2016). RNNs consist of three layers: an input layer, a hidden layer, and an output layer. RNNs handle sequential data better than feed-forward networks because the hidden layer receives both current inputs at time step *t* as well as information about its previous hidden state at time step *t*−1 through a recurrent connection as following: *h*(*t*) = θ(*x*_*t*_, *h*_*t*−1_). The function θ is a non-linearity such as tanh or sigmoid. Unlike other types of neural networks, RNNs share the same parameters across all steps, thus, reducing the number of parameters the networks need to learn. However, training RNNs can be complicated by vanishing and exploding gradients (Bengio et al., 1994), which greatly affects long-term dependencies learning. To address these problems, several variants of RNNs such as GRU (Cho et al., 2014) or LSTM (Schmidhuber and Hochreiter, 1997) have been proposed.

We implement our proposed method using GRU. We choose GRU because of the nature of our dataset; the length of the time series covers up to 50 observations. GRU is computationally more efficient than the commonly used LSTM because it provides a simpler network but with a comparable performance (Chung et al., 2014). Particularly, GRU addresses the vanishing gradient problem as well as capture the effect of long-term dependencies by applying the gating mechanism. The GRU architecture updates hidden states using the following equations:


(1) $$\begin{array}{r} z_{t}=\sigma \left(W_{z}x_{t}+U_{z}h_{t-1}+b_{z}\right) \end{array}$$

(2) $$\begin{array}{r} r_{t}=\sigma \left(W_{r}x_{t}+U_{r}h_{t-1}+b_{r}\right) \end{array}$$

(3) $$\begin{array}{r} \tilde{h_{t}}=\tanh\left(W_{h}x_{t}+U_{h}\left(h_{t-1}⊙r_{t}\right)+b_{h}\right) \end{array}$$

(4) $$\begin{array}{r} h_{t}=z_{t}⊙h_{t-1}+\left(1-z_{t}\right)⊙\tilde{h_{t}} \end{array}$$

where *z*_*t*_ is the vector for update gate with weight matrices *W*_*z*_ and *U*_*z*_; *r*_*t*_ is the vector for resetting the gate with weight matrices *W*_*r*_ and *U*_*r*_; σ denotes the sigmoid function and ⊙ denotes the element wise multiplication; *x*_*t*_ is the input at time step *t*, *h*_*t*−1_ is the previous hidden state; *b*_*z*_, *b*_*r*_, and *b*_*h*_ are trainable bias vectors. In this architecture, the update gate selects whether the hidden state is updated with a new hidden state $\tilde{h_{t}}$ while the reset gate decides whether the previous hidden state *h*_*t*−1_ is ignored (Cho et al., 2014).

We train each RNN using the Adam optimizer (Kingma and Ba, 2014) and mean squared error as a loss function. To combat exploding gradients, we apply weight decay. Additionally, we use Exponential Smoothing (Brown, 1959) to smooth the forecast time series. With the sequence of observations with start time at *t* = 0, the exponential smoothing function is defined by the following formulas: *s*_0_ = *x*_0_ and *s*_*t*_ = α*x*_*t*_+(1−α)*s*_*t*−1_, where *s*_*t*_ is the output of the algorithm and *x*_*t*_ is the actual observation at time *t*; and 0 < α <1 is the smoothing factor.

We use mean absolute percentage error (MAPE), unbiased MAPE (uMAPE), and volume-weighted MAPE (vMAPE) to measure the prediction accuracy of a forecasting series. We use the following formulas to calculate the error where *x*_*t*_ is the actual value and *y*_*t*_ is the forecast value at time *t*:


(5) $$\begin{array}{r} MAPE=\frac{100\%}{n}\overset{n}{\underset{1}{\sum }}|\frac{y_{t}-x_{t}}{x_{t}}| \end{array}$$

(6) $$\begin{array}{r} uMAPE=\frac{100\%}{n}\overset{n}{\underset{1}{\sum }}|\frac{y_{t}-x_{t}}{\left(x_{t}+y_{t}\right)/2}| \end{array}$$

(7) $$\begin{array}{r} vMAPE=\frac{\overset{n}{\underset{1}{\sum }}|y_{t}-x_{t}|}{\overset{n}{\underset{1}{\sum }}|x_{t}|} \end{array}$$

During training, for each model, we compute three error evaluation metrics (MAPE, uMAPE, vMAPE). We choose the model that scores best on at least two of the three validation metrics. If there is a tie, we choose the model with the best vMAPE. Thus network architectures and parameters are chosen based on validation performance.

### 3.2. SNN Architecture

Spiking neural networks (SNNs) are inspired by information processing in biology, where sparse and asynchronous binary signals are communicated and processed in a massively parallel fashion. SNNs are the third generation of neural networks (Maass, 1997), in which neurons communicate through binary signals known as spikes. An SNN architecture consists of spiking neurons and interconnecting synapses that are modeled by adjustable scalar weights. SNNs are capable of learning rich spatio-temporal information (Kasabov, 2014).

Training for classification models of SNNs has recently been investigated based on both the supervised and unsupervised mechanisms. In this work, we use the adapted Widrow-Hoff learning rule proposed in Ponulak and Kasiński (2010). The learning rule for the *ith* synaptic input to neuron *n* is given by:


(8) $$\begin{array}{r} \Delta w_{ni}=\left(y^{d}-y^{o}\right)*x_{i} \end{array}$$

where *w*_*ni*_ is the amount of weight change for the synapse, *x*_*i*_ is the *i*^*th*^ synaptic input, *y*^*d*^ and *y*^*o*^ are the desired and observed outputs, respectively.

We use simulated networks of leaky integrate-and-fire (LIF) neurons in the experiments, which is the most popular one for building SNNs. LIF neurons are characterized by the internal state called the membrane potential. The membrane potential integrates the inputs over time and generates an output spike when the neuronal firing threshold. As mentioned above, there is a positive association between mRNA and protein levels, thus different expression levels should correspond to different mRNA abundance patterns. Consequently, when these expressions with different patterns are converted into spikes in the SNN, the membrane voltage of each output spiking neuron would be different. Additionally, it has been shown that the SNN can achieve a better performance of classification if the membrane voltages of output spiking neurons vary from each other (Yu et al., 2014). Therefore, we implemented SNN with LIF because it is computationally simple, can easily be implemented in any hardware, and appropriated for our problem.

The operation of the LIF neuron model is described by five basic operations: synaptic integration, leak integration, threshold, spike firing, and reset (Cassidy et al., 2013).


(9) $$\begin{array}{r} V_{t}=V_{t-1}+\overset{N-1}{\underset{i=0}{\sum }}x_{i}\left(t\right)s_{i} \end{array}$$

(10) $$\begin{array}{r} V_{t}=V_{t}-\lambda \end{array}$$

(11) $$\begin{array}{r} If\;V_{t}\geq \alpha \;then\;Spike\;and\;V_{t}=R \end{array}$$

where *V*_*t*_ is the membrane potential, *t* is discrete time step, *N* is the number of synapses, *x*_*i*_(*t*) is the *ith* synapse, *s*_*i*_ synaptic weight of *ith* synapse, λ is leak, α is spiking threshold, and *R* is the resting potential. During the integration, for each neuron in a time step, the membrane potential is the sum of the membrane potential in the previous time step and the synaptic input. Following the integration, the model subtracts the leak value from the membrane potential, and finally, when the membrane potential reaches a threshold, the neuron spikes and the membrane potential is reset to a resting value.

The mRNA abundance information is presented to the SNN directly without significant transformation. We directly mapped the time series values onto distinct neurons so that temporal information of the time series is encoded directly into the network without extra processing. We use receiver operator characteristic (ROC) curves, and the prediction accuracy to evaluate each model. The area under the ROC curve (AUC) quantifies the ability of the classifier to balance sensitivity (true positives) against specificity (avoiding false positives).

### 3.3. Dataset

We generate a list of genes corresponding to those potential drug targets from Fatumo et al. (2009) and our previous work in Tran and Ekenna (2017). To broaden our dataset, we used PlasmoDB (Aurrecoechea et al., 2008) to find all the related genes to our list based on protein sequence similarities via utilization of the P-BLAST tool (Altschul et al., 1990). We acquire mRNA abundance information for those genes from Painter et al. (2018). Finally, we normalize (from −1 to 1) the mRNA abundance for each gene using the following equation:


(12) $$\begin{array}{r} X_{norm}=-1+\frac{2*\left(X-X_{min}\right)}{X_{max}-X_{min}} \end{array}$$

We apply the normalization method, so the training process is more stable and faster since it's shown that gradient descent converges much faster with feature normalization than without it (Ioffe and Szegedy, 2015). The dataset contains 697 genes and their mRNA abundances are represented with a time series of 48 observations. We split the dataset into the set for training, validation, and testing. For this work, we group the 48 h period into three main asexual stages within the red blood cell cycle: ring (0–21 hpi), trophozoite (22–32 hpi), and schizont (33–47 hpi). We combine the mRNA abundance information from Painter et al. (2018) and the expression level from Das et al. (2019) to create the additional dataset for testing our classification method. Overall the dataset contains 1991 genes with their normalized mRNA abundance and expression level. For genes expression level, we split the 48 h period into six stages: early ring, late ring, early trophozoite, late trophozoite, early schizont, and late schizont.

## 4. Experimental Results

### 4.1. Evaluation Methods

#### 4.1.1. Time Series Prediction

In this work, we are interested in forecasting the mRNA abundance during the parasite life cycle in the blood stage. Thus we use the information from the previous stage to forecast the next stage. Specifically, we implement two RNN models to perform three experiments since we have different lengths for time series for each experiment.

1. **Experiment 1:** using time series of ring stage to predict time series of trophozoite stage;
2. **Experiment 2:** using time series of both ring stage and trophozoite stage to predict time series of schizont stage;
3. **Experiment 3:** using time series of ring stage to predict time series of trophozoite stage then using the combining time series to predict the time series of schizont stage.

RNN1 is used for experiment 1, and RNN2 is used for both experiment 2 and 3. Each experiment was repeated 10 times and the average performance is reported.

To evaluate our method's performance, we compared it to classical time series forecasting methods such as Auto-regressive Moving Average (ARMA) (Hamilton, 1994), Simple Exponential Smoothing (SES) (Brown, 1959), and Holt Winter's Exponential Smoothing (HWES) (Winters, 1960). We utilized and implemented these methods because they are proven to be efficient in time series data forecasting especially considering the nature of our dataset which is univariate time series data without seasonality.

#### 4.1.2. Genes Expression Prediction

In this work, we will predict the expression level for each stage during the parasite life cycle. Therefore, we use the time series of mRNA abundance to predict the expression level, which is either low or high, for the ring, trophozoite, and schizont stage. Specifically, we implement the SNN model to perform the following experiment: using time series of 48 h periods to predict the expression level for each: late ring (LR); late trophozoite (LT); late schizont (LS). We use the whole time series of 48 h periods to predict instead of its average, minimum, or maximum values because we want to keep the time aspect of the mRNA abundance. Additionally, with little input data, the machine learning model is very likely to be under-fitting, in which it is unable to capture the relationship between the input and output variables accurately.

To evaluate our method's performance, we compared it to state-of-art models used in Read et al. (2019): logistic regression with elastic net regularization (LogR), tree model with gradient boosting (TGB), and multi-layer perceptron model (MLP). Since the work in Read et al. (2019) only predict the expression level for the late ring; the late trophozoite; the late schizont, we designed the experiment to compare our model performance with them. The experiment was repeated 10 times and the average performance is reported.

### 4.2. Implementation

Our method is implemented using Python with Pytorch (Paszke et al., 2019). For each model, we used 70% of the dataset to train and 20% of the data set to test. The remaining 10% is used as a validation set to optimize the model parameters. For RNN, we implemented them with two hidden layers. The first hidden layer has the same size as the input layer and the second hidden layer the same as the output layer size. We trained both RNNs using a batch size of 1, learning rate of 0.01, and weight decay values of 10^−5^. The number of epoch and smoothing factors are 200, 300, and 0.5, 0.4 for RNN1 and RNN2, respectively. We use the provided statistics library in Python to implement ARMA, SES, and HWES.

We implement SNN using Python with PyTorchSpiking (Research, 2020). Similar to RNN, we used 70% of the dataset to train and 20% of the data set to test, and 10% is used for validation. We implemented SNN with one hidden layer, which has half the size of the input layer. We simulated SNN for 10 s, the maximum firing rate of the input neurons is 100 Hz, the batch size is 100, the number of epoch is 200, and the default value for other parameters. We used the provided information from Read et al. (2019): LogR using the scikit-learn implementation (Pedregosa et al., 2011), TGB using the XGboost Python implementation (Chen and Guestrin, 2016), and MPL using Pytorch with two hidden layers, each containing the same number of nodes as the input layer.

### 4.3. Results

#### 4.3.1. Time Series Plot Prediction Results

Table 1 shows the performance of our proposed model comparing to other time series forecasting methods. The best performing method is highlighted in bold. Since our evaluation metric measures the error rate, the lower the error values are, the better the forecasting series are. Overall, our method has the best performance because it models the non-linear relationship between each observation while is lacking in other comparable methods.

**Table 1:** Comparison among forecasting methods.

	**Experiment 1**			**Experiment 2**			**Experiment 3**		
	**MAPE**	**uMAPE**	**vMAPE**	**MAPE**	**uMAPE**	**vMAPE**	**MAPE**	**uMAPE**	**vMAPE**
Our method	**0.74**	**1.05**	**0.17**	2.06	**0.90**	**0.15**	2.96	**3.62**	**0.22**
ARMA	0.91	8.74	0.38	2.50	4.25	0.39	**1.52**	9.12	0.62
SES	1.50	3.58	0.31	**1.54**	7.51	0.29	4.98	21.3	0.77
HWES	1.15	1.75	0.34	2.69	56.0	0.36	4.13	7.63	0.72

To better show the performance of our method, forecast time series are deemed accurate if one of their calculated error rates is less than a certain threshold. Table 2 shows the overall accuracy of our proposed model with 10, 20, and 30% error rates when forecasting mRNA abundance time series. For experiment 1, our results indicated that 87% of the forecast time series have a 70% similarity with the actual time series; 72% of the forecast time series have a 80% similarity; and 39% of the forecast time series have a 90% similarity. For experiment 2, 92, 78, and 45% of the forecast time series have a 70, 80, and 90% similarity, respectively. For experiment 3, 77, 56, and 26% of the forecast time series have a 70, 80, and 90% similarity, respectively.

**Table 2:** Accuracy of our model.

**Error rate**	**10 (%)**	**20 (%)**	**30 (%)**
Experiment 1	38.57	72.38	87.14
Experiment 2	45.44	77.89	91.70
Experiment 3	25.85	56.46	77.21

During our experiments, we observed that the more time-series information we use as input, the more accurate our forecast. Experiment 3 has a slightly lower accuracy because errors accumulate from forecasting earlier time series for the trophozoite stage to forecasting time series for the schizont stage. Overall, the accuracy of our predicted results is high.

#### 4.3.2. Time Series Plot Results

**Figure 2:**
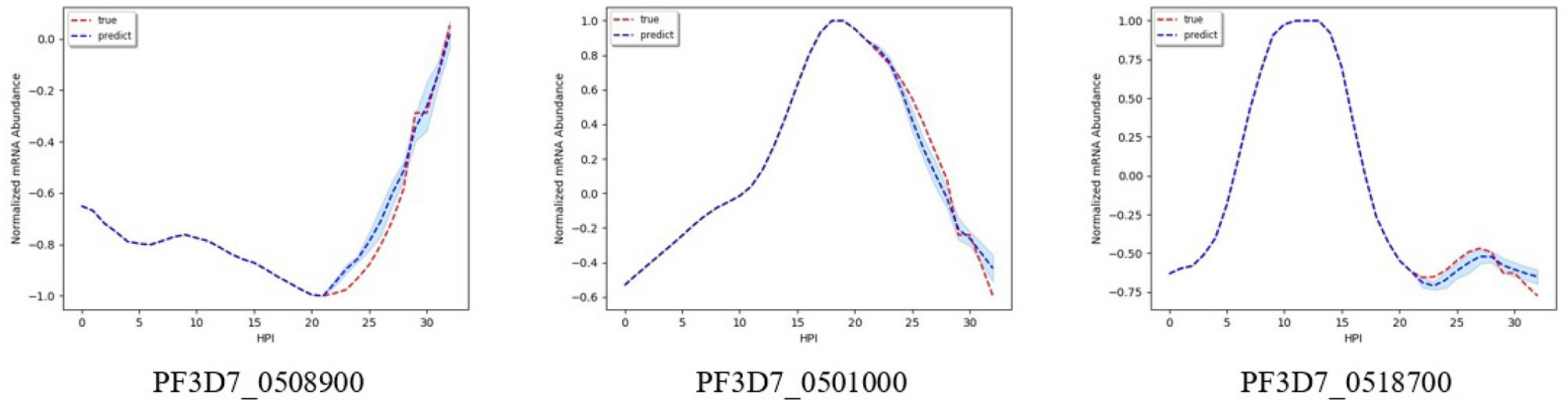
Examples of results for experiment 1.

**Figure 3:**
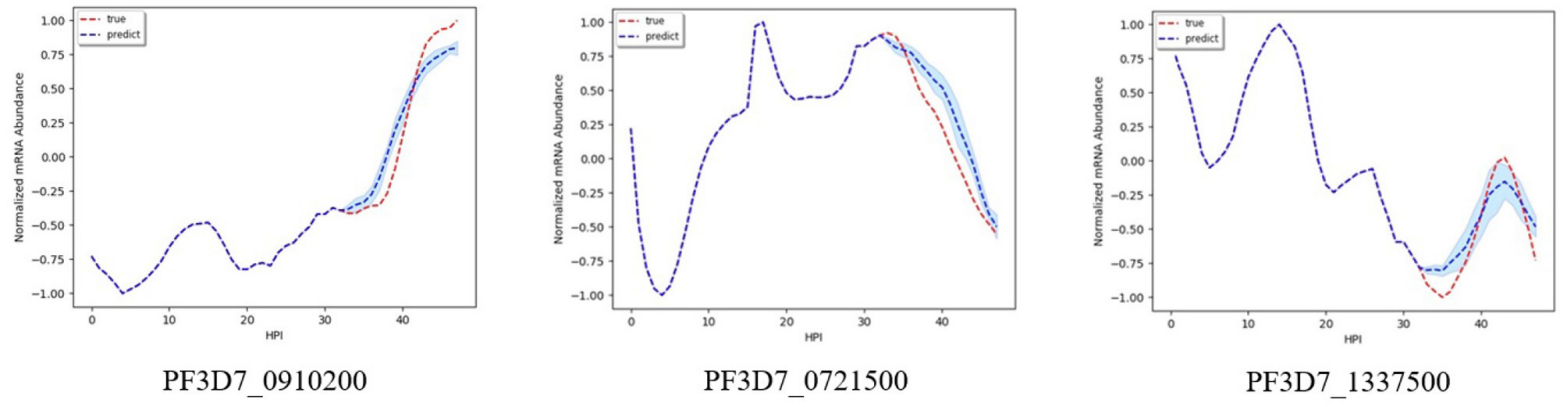
Examples of results for experiment 2.

**Figure 4:**
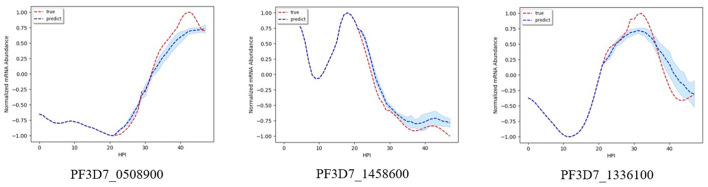
Examples of results for experiment 3.

**Figure 5:**
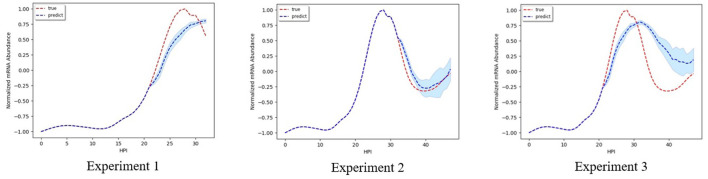
Forecast series for Dihydrolipoyl dehydrogena.

Figures 2–**5** shows time-series results on some sample genes when using our method to forecast different gene expression profiles. The red line represents the actual time series from experimental data, the blue line represents the average forecast time series, and the shaded blue region represents its standard deviations over ten runs using our model. We present genes in this paper that illustrate how our forecast time-series can follow the expression profile (upward trend, downward trend, or both).

Figure 2 shows results on the three different genes for experiment 1. Both the magnitude and trend of the forecast time series follow closely the actual time series gotten from experimental data, with a very small standard deviation (blue shaded region). Figure 3 shows the results on the three different genes for experiment 2. Similar to the procedure taken in experiment 1, the forecast time series also follows closely to the actual time series, with also very small standard deviation, showing the robustness of our method to predict the gene expression profile. Figure 4 shows the results on three different genes for experiment 3. Although the forecast time series is not as accurate and the standard deviation is larger, it still follows the trend of the actual time series. Thus, we are still able to determine the gene expression profile reliably.

Figure 5 shows the result when using our model to predict the mRNA abundance profile for genes PF3D7_1232200 associated with Dihydrolipoyl dehydrogena. Dihydrolipoyl dehydrogena is one of the enzymes we identified as a potential drug target from our previous work (Tran and Ekenna, 2017) using knock-out reactions in the metabolic network model. We illustrate how our model can forecast the genes expression profile. The accuracy of the forecast time series is high, especially in experiment 2. In experiments 1 and 3, the magnitude of the forecast mRNA abundance is different from the actual one, but the upward and downward trend in genes expression profile is identical. Thus, we are still able to determine at which stage the genes are the most active.

#### 4.3.3. Genes Expression Prediction Results

Table 3 shows the performance of our proposed model comparing to other prediction methods. The best performing method is highlighted in bold. We evaluate our model using an accuracy rate. For MLP and SNN, the accuracy is averaged over 10 trials. Overall, our method has the best performance.

**Table 3:** Comparison among classification methods.

	**LogR**	**TGB**	**MLP**	**SNN**
LR	0.802	0.779	0.766	**0.836**
LT	0.899	0.902	0.895	**0.910**
LS	0.758	0.771	0.792	**0.815**

**Figure 6:**
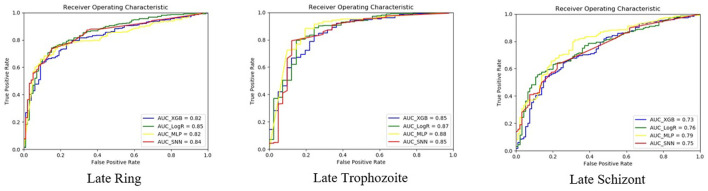
ROC of classification models for each stage.

Figure 6 shows the ROC curves when using the time series from the test set to classify the expression level for the late ring, late trophozoite, and late schizont. We reported the trial with the median accuracy for all the comparable methods. The AUCs of our method are 0.84, 0.85, and 0.75 for the late ring, the late trophozoite, and the late schizont. Overall, we observed similar levels of performance across the three erythrocytic stages.

Overall, our model forecasts the time series accurately, the trend of expression for each gene is closely captured by our model, and the expression levels are correctly classified. Additionally, with our current dataset, it takes only a few minutes to train our models, and a few seconds to forecast the time series and classify the expression level. Genes which highly express during the trophozoite stage produce proteins increasing the metabolic activity of the parasite. Thus, by accurately forecasting the gene expression profiles, we can verify whether the genes are active during the trophozoite stage. Moreover, the genes which highly express during the schizont stage, are related to the replication and mitotic division of the parasite. Knowing which genes express during the schizont stage, could potentially aid in better knowledge and control over the parasite development cycle. Finally, since the model could forecast the time series and expression level for the trophozoite and schizont stage, it could potentially reduce time and serve as an important template for biologists. Therefore, with our model's high accuracy, we can potentially provide a beneficial tool to help biologists generate a model of the complete gene expression profiles even before performing more involving experiments in the lab. In some cases where there is an abundance of data but there could be some missing data at some time point for some reason, our work could fill in those gaps by accurately predicting the missing data.

## 5. Conclusion

In conclusion, to our knowledge, we are the first one to proposed the usage of RNNs and SNNs to forecast the gene expression profile for the *P. falciparum* parasite during the blood-stage life cycle. Even with a small dataset for training, our model performs very well in most scenarios. Overall, our method can forecast adequately the magnitude, the trend of mRNA abundance, and the expression level of the *P. falciparum* parasite during the blood stage life cycle. In the future, with the forecast gene profiles, we would like to predict the relationship among those genes to identify the gene or group of genes which express potential drug target enzymes. Considering the many existing variants of the *Plasmodium* parasite, we plan to evolve our method to investigate the profiles of these variants. Additionally, we plan to refine our model so that it will take a smaller amount of data (3–5 data points) and predict the next immediate data point (instead of the whole next stage), thus making it more practical for biologists.

## Data Availability Statement

Publicly available datasets were analyzed in this study. This data can be found at: https://www.nature.com/articles/s41467-018-04966-3.

## Author Contributions

TT and CE contributed to the design of the recurrent neural network. BR contributed to the design of the spiking neural network. TT provides the implementation of the study and wrote the first draft. BR and CE revised the draft. All authors contributed to manuscript revision, read, and approved the submitted version.

## Conflict of Interest

The authors declare that the research was conducted in the absence of any commercial or financial relationships that could be construed as a potential conflict of interest.

## Publisher's Note

All claims expressed in this article are solely those of the authors and do not necessarily represent those of their affiliated organizations, or those of the publisher, the editors and the reviewers. Any product that may be evaluated in this article, or claim that may be made by its manufacturer, is not guaranteed or endorsed by the publisher.
